# Minimum Radiant Exposure and Irradiance for Triggering Adequate Polymerization of a Photo-Polymerized Resin Cement

**DOI:** 10.3390/ma14092341

**Published:** 2021-04-30

**Authors:** Qi Li, Hong-Lei Lin, Ming Zheng, Mutlu Ozcan, Hao Yu

**Affiliations:** 1 Fujian Key Laboratory of Oral Diseases, Fujian Provincial Engineering Research Center of Oral Biomaterial, Stomatological Key Laboratory of Fujian College and University, School and Hospital of Stomatology, Fujian Medical University, Fuzhou 350000, China; linhonglei@fjmu.edu.cn (Q.L.); linhonglei@foxmail.com (H.-L.L.); 2 Fujian Provincial Governmental Hospital, Fuzhou 350000, China; 3 Division of Dental Biomaterials, Clinic for Reconstructive Dentistry, Center for Dental Medicine, University of Zurich, 8006 Zurich, Switzerland; mutluozcan@hotmail.com

**Keywords:** radiant exposure, irradiance, polymerization, photo-polymerized resin cement

## Abstract

This study aimed to establish the minimum radiant exposure and irradiance to trigger an adequate polymerization of a photo-polymerized resin cement. In total, 220 disc-shaped specimens (diameter of 10 mm and thickness of 0.1 mm) were fabricated using a photo-polymerized resin cement (Variolink N-transparent, Ivoclar Vivadent). To investigate the minimum radiant exposure, the specimens were polymerized with radiant exposures of 1, 2, 3, 4, 5, 6, and 18 J/cm^2^ (*n* = 20). During polymerization, the irradiance was maintained at 200 mW/cm^2^. To investigate the minimum irradiance, the specimens were polymerized with irradiances of 50, 100, 150, and 200 mW/cm^2^ (*n* = 20). During polymerization, the radiant exposure was maintained at the previously determined minimum radiant exposure. The Vickers microhardness (HV) and degree of conversion (DC) of the carbon double bond of the specimens were measured to determine the degree of polymerization of the specimens. The results were analyzed using one-way analysis of variance (ANOVA) and Tukey’s test (*p* < 0.05). In the investigation of the minimum radiant exposure, the HV and DC of the specimens polymerized with a radiant exposure from 1 to 5 J/cm^2^ were significantly lower than those with 18 J/cm^2^ (all *p* < 0.05). However, no significant difference in HV and DC was found between the specimens polymerized with 6 J/cm^2^ and 18 J/cm^2^ (*p* > 0.05). In the investigation of the minimum irradiance, the specimens polymerized with an irradiance of 50 mW/cm^2^ had significantly lower HV and DC than the specimens polymerized with an irradiance of 200 mW/cm^2^ (*p* < 0.05). However, no significant difference in the HV and DC was found among the specimens cured with irradiances of 100, 150, and 200 mW/cm^2^ (*p* > 0.05). In conclusion, the minimum radiant exposure and irradiance to trigger an adequate polymerization of the light-cured resin cement were 6 J/cm^2^ and 100 mW/cm^2^, respectively.

## 1. Introduction

All-ceramic restorations are increasingly preferred by dentists and patients because of their natural appearance, biocompatibility, durability, and high compressive resistance [1]. A high bond strength for the adhesion complex formed between the ceramic, resin cement, and dental hard tissues is vital for the success of a ceramic restoration [2]. Inadequate polymerization of resin cements leads to microleakage, poor retention of the restoration, poor marginal adaptation, easy fracture, unstable color, and cytotoxicity [3,4,5,6].

Resin cements are classified into three types according to their activation modes: chemical-cured (CC), photo-polymerized/light-cured (LC), and dual-cured (DC) resin cements [7]. LC resin cements are most used in aesthetic rehabilitation due to their better color stability than that of the other cements [7]. The polymerization of LC resin materials is dependent on the total energy of the irradiation, e.g., the radiant exposure (RE), which is the product of the irradiance (mW/cm^2^) (also known as light intensity) and irradiation time [8]. Different combinations of irradiance and light curing time provide similar material properties at constant REs, which is known as the exposure reciprocity law (ERL) [9].

In dental practice, LC resin composites are used to restore dental defects, and LC resin cements are used to bond the dental restoration. The REs required to adequately polymerize 1.5 and 2 mm thick LC resin composites were reported to be 18 and 24 J/cm^2^, respectively [9,10,11]. Moreover, a rating scale developed by the manufacturer (Demetron/Kerr, CA, USA) showed that the polymerization from 2 to 3 mm thick LC resin composites would be inadequate when the irradiance is below 200 mW/cm^2^ [12]. Although resin cements have a similar composition as resin composites [13], the thickness of resin cements in clinical applications is much thinner (~0.1 mm) [14,15]. Therefore, it is conceivable that the RE and irradiance requirements would be different for LC resin cements. However, limited information is available in the literature.

Although the irradiance of contemporary light-emitting diode (LED) light curing units (LCUs) is commonly far higher than the abovementioned threshold (200 mW/cm^2^) [16], the actual irradiance reaching the LC resin cement is reduced drastically during light curing through a ceramic restoration. A previous study reported that the irradiance decreased by >80%, 95%, and 99% through 1.5, 3.0, and 6.0 mm thick glass ceramic discs, respectively, compared to the irradiance with no ceramic disc at 0 mm distance [16]. Considering such massive attenuation of the irradiance, it is necessary for dentists to know the minimum required irradiance and required RE to adequately polymerize an LC resin cement, which were the purposes of this study.

## 2. Materials and Methods

**Figure 1:**
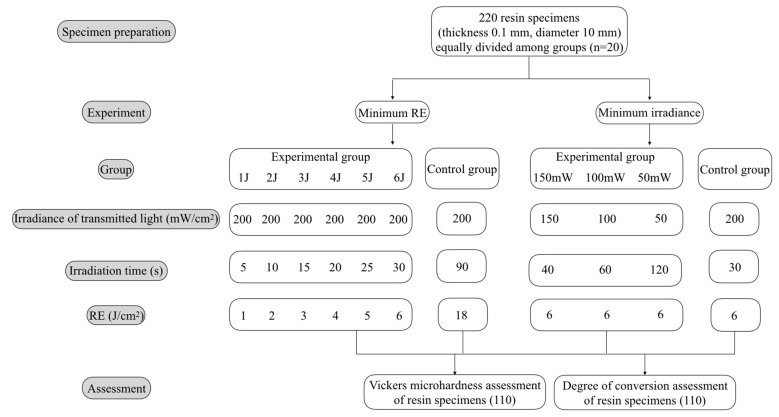
Flow diagram for this study.

The experimental protocol is exhibited in Figure 1.

### 2.1. Specimen Preparation

An LED LCU (Bluephase N, Ivoclar Vivadent, Liechtenstein) was used in the present study. The actual irradiance of the LED LCU (700 mW/cm^2^) was determined by an optical power meter (Sensor S1350C, Console PM100D, Thorlabs, Newton, NJ, USA). Four glass ceramic plates (Empress HT, Ivoclar Vivadent, Schaan, Liechtenstein) with different thicknesses (1, 1.5, 2, and 3 mm; light attenuation rate: 71.5, 78.5, 86, and 93%) were used to attenuate the irradiance of the light from the unit at 200, 150, 100, and 50 mW/cm^2^.

**Figure 2:**
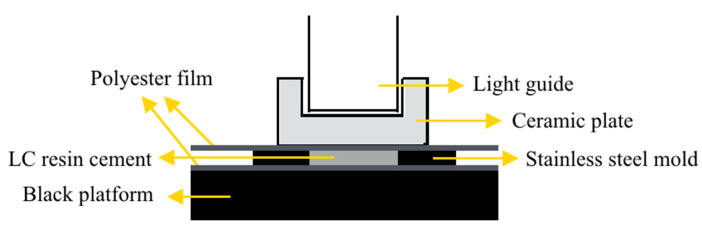
Polymerization apparatus for the LC resin cement.

The specimens were fabricated with a customized stainless-steel mold (thickness of 0.1 mm and internal diameter of 10 mm) using a LC resin cement (Variolink N-transparent, Ivoclar Vivadent, Liechtenstein). An appropriate amount of the resin cement was applied to the stainless-steel mold. Light curing was performed by placing the tip of the LCU in contact with the ceramic plate. A black countertop was used to support the stainless-steel mold with the specimen and decrease the light reflectivity [17]. A polyester film was placed on the top and bottom of the specimen to isolate it from the glass ceramic plate and black countertop and prevent oxygen-inhibition during polymerization [7,17] (Figure 2).

### 2.2. Determination of the Minimum RE

The software G*power (version 3.1, Dusseldorf, Germany) was used to calculate the number of specimens. The power for the primary outcome (surface microhardness) was calculated based on a two-sided *t*-test with a significance level of 5% and a statistical power of 80%. The results indicated that a minimum sample size of 10 was required per group.

The experiment was performed in a darkroom environment. Based on a previous study [12], 200 mW/cm^2^ was considered adequate to trigger polymerization of the 0.1 mm thick LC resin cement specimens. Six experimental groups (1 J, 2 J, 3 J, 4 J, 5 J, and 6 J) and 1 positive control group were adopted according to the RE (*n* = 20). According to previous studies [9,11], 18 J/cm^2^ was selected in the positive control group. The transmitted light from 200 mW/cm^2^ was irradiated onto the specimens from groups 1 J, 2 J, 3 J, 4 J, 5 J, 6 J, and the control for 5, 10, 15, 20, 25, 30, and 90 s, providing REs of 1, 2, 3, 4, 5, 6, and 18 J/cm^2^, respectively. A digital microhardness tester (HXD-1000TM/LCD, Baoling, China) was used to produce 3 microindentations (1 at the center and others at ≥1 mm from the center) on the upper surface of each specimen. The microindentations were then measured to determine the Vickers microhardness (HV) of the specimens (*n* = 10). Measurements were performed under a load of 50 gf for 15 s [17,18,19]. A Fourier transform infrared spectrometer (Nicolet iS50, Thermo Scientific, Waltham, WJ, USA) was used to test the degree of conversion (DC) of carbon double bond of the specimens (*n* = 10). The absorbance spectra of uncured resin materials and cured resin specimens were acquired by scanning the specimens 32 times over a 4000–400 cm^−1^ range with a resolution of 4 cm^−1^ (OMNIC spectra Software, version 6.2, Thermo Scientific, Madison, WI, USA). The DC was calculated from the absorbance intensity of aliphatic carbon double bond peaks (1638 cm^−1^) and the ratio of the absorbance intensity of aromatic carbon double bond peaks (1608 cm^−1^) of cured (C) and uncured (U) resin. DC was calculated as follows: DC (%) = [1 − (1638 cm^−1^/1608 cm^−1^)_Peak height after curing_/(1638 cm^−1^/1608 cm^−1^)_Peak height before curing_] × 100 [16,20]. All data were collected at 25 °C ± 1 °C and 60 ± 5% humidity conditions and shielded from ambient and room light. Before the measurements, all specimens were placed in the dark for 15 min [21]. According to the statistical analysis, when the mean value of HV and DC of certain experimental groups did not have significantly different mean values of HV and DC from that of the control group, the minimum RE of the experimental groups was considered the minimum RE to trigger an adequate polymerization of the resin cements.

### 2.3. Determination of the Minimum Irradiance

The experiment was performed in a darkroom environment. Three experimental groups (50 mW, 100 mW, and 150 mW) and 1 positive control group were adopted according to the irradiance (*n* = 20). The transmitted light from 50, 100, 150, and 200 mW/cm^2^ was irradiated to the specimens from the groups 50 mW, 100 mW, 150 mW, and a positive control for a certain period of time (200 mW/cm^2^ for 30 s) to provide the previously determined minimum RE. The HV and DC of the specimens were measured as described above. According to the statistical analysis, when certain experimental groups did not have significantly different mean HV and DC from the control group, the minimum irradiance of the experimental groups was considered the minimum irradiance to trigger an adequate polymerization of the resin cements.

### 2.4. Statistical Analysis

Statistical analysis was performed using the SPSS 25.0 software package (Armonk, NY, USA). Shapiro-Wilk test was used to test the data normality. One-way analysis of variance (ANOVA) and Tukey’s test were performed on the HV and DC. *p* < 0.05 was considered to be statistically significant.

## 3. Results

**Figure 3:**
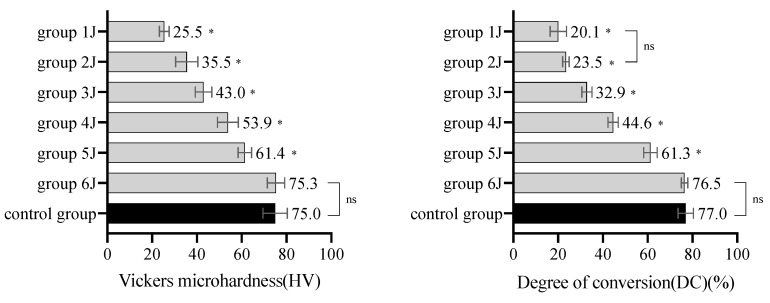
HV and DC of the LC resin cements triggered by different REs in the minimum RE experiment (mean with SD, *n* = 10). * indicates that the HV or DC of the group was significantly different from that of the control group (*p* < 0.05). “ns” indicates that there was no significant difference in the HV or DC between two groups (*p* > 0.05).

In the minimum RE experiment, all mean HV and DC values of the specimens in groups 1 J, 2 J, 3 J, 4 J, and 5 J were significantly different from those in the control group (all *p* < 0.05). However, no significant difference in mean HV and DC was found between group 6J and the control group (*p* > 0.05) (Figure 3). Therefore, 6 J/cm^2^ was determined as the minimum RE to trigger an adequate polymerization of the resin cements.

**Figure 4:**
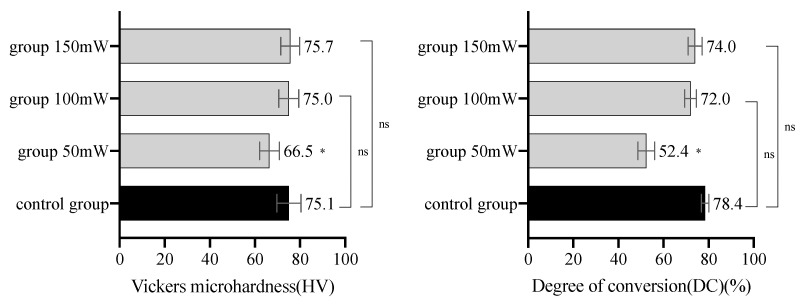
HV and DC of the LC resin cements triggered by different irradiances in the minimum irradiance experiment (mean with SD, *n* = 10). * indicates that the HV or DC of the group was significantly different from that of the control group (*p* < 0.05). “ns” indicates that there was no significant difference in the HV or DC between two groups (*p* > 0.05).

In the minimum irradiance experiment, the specimens in the 50 mW group had significantly different mean values of HV and DC from those in the control group (*p* < 0.05). The specimens in the 150 mW and 100 mW groups did not have significantly different mean values of HV and DC from those in the control group (all *p* > 0.05) (Figure 4). Therefore, 100 mW/cm^2^ was determined as the minimum irradiance to trigger adequate polymerization of the resin cements. 

## 4. Discussion

When an LCU irradiates a resin cement through a ceramic restoration, the actual irradiance reaching the cement is reduced to a certain extent due to the attenuation effect of the ceramic on the light, and the polymerization of the cement may be compromised [16]. Although previous studies have been performed regarding the adequate polymerization of LC resin composites for restorative purposes (filling) [10,11,12], information regarding the adequate polymerization of LC resin cements for bonding is limited. The present study, for the first time, investigated the minimum irradiance and RE required to adequately polymerize an LC resin cement.

The extent of polymerization of resin cements can be measured by several methods. Fourier transform infrared spectroscopy (FTIR) can directly measure the extent of polymerization of a resin by assessing the DC of the carbon double bond, which evaluate the change in ratio of characteristic absorbance peaks of the cured and uncured resin. Commonly, the aliphatic carbon double bond peak height or area at 1638 cm^−1^ is compared to the aromatic carbon double bond peak height or area at 1608 cm^−1^ [9,16,20]. Furthermore, the microhardness has been shown to be a simple and reliable indicator of the carbon double bond conversion, and it has been used to indirectly measure the extent of polymerization of resins [22,23,24]. Therefore, the DC and microhardness test were used in the present study. Previous studies on the internal fit and marginal adaptation of restorations showed that a gap between a restoration and a tooth ranged was tens to hundreds of microns [14,15,25,26]. Therefore, the cement thickness of the polymerization model was set as 0.1 mm in this study.

In the present study, the minimum RE and irradiance to trigger an adequate polymerization of the LC resin cement were 6 J/cm^2^ and 100 mW/cm^2^, respectively, which is obviously different from the previous results for an LC resin composite (18 J/cm^2^ and 200 mW/cm^2^ for a 1.5 mm thick LC resin composite) [9,10,11]. This difference may be due to the different thicknesses of the resin cement and composite. Moreover, the scraping test was used in a previous study regarding LC resin composites [11]. However, for LC resin cements with a thickness of 0.1 mm, it is difficult to perform the scraping test. In accordance with previous studies, the DC and microhardness test were adopted.

According to a previous study [16], when light curing the LC resin cement through 1.5, 3.0, and 6.0 mm ceramic restorations, the light attenuation rates were >80%, 95%, and 99%, respectively. Theoretically, to achieve the minimum irradiance of 100 mW/cm^2^ and RE of 6 J/cm^2^ established in the present study, the irradiance of the LCU should be at least 500, 2000, and 10,000 mW/cm^2^ when light curing the LC resin cement through 1.5, 3, and 6 mm ceramic restorations, respectively, and the irradiation time should be more than 60 s. However, the irradiance of most commercially available LCUs is below 2000 mW/cm^2^ [16,17,27,28]. Therefore, LC resin cement should not be used when the ceramic restorations exceed 3.0 mm in thickness.

The results of this study also tested the effectiveness of the ERL. In the present study, the specimens were inadequately polymerized when the irradiance was 50 mW/cm^2^, regardless of the irradiation time of 120 s. This apparent violation of the ERL is similar to what occurred in previous studies. Although the RE plays an important role, the irradiance and irradiation time independently affect the polymerization of resin [29,30,31,32,33]. Therefore, both time and irradiance should be considered critical in the polymerization process.

The minimum irradiance and RE values should be 50–100 mW/cm^2^, and 5–6 J/cm^2^, respectively. However, the samples were not tested at these experimental settings due to technical limitation, which was a limitation of the present study. Furthermore, the cement thickness and resin cements may vary in clinical conditions [34]. Only 1 LC resin cement with a thickness of 0.1 mm was tested in the present study, which may be considered another limitation. Therefore, the present findings should be interpreted with caution. Future studies are required to evaluate different types of resin cements.

## 5. Conclusions

From this study, it can be concluded that the minimum RE and irradiance to trigger an adequate polymerization of a 0.1 mm thick photo-polymerized resin cement are 6 J/cm^2^ and 100 mW/cm^2^, respectively.

## Data Availability

Further data may be requested by contacting the corresponding author. We declare that any data regarding the study will willingly provided.

## References

[1] Borges G., Agarwal P., Miranzi B., Platt J., Valentino T., dos Santos P. (2008). Influence of different ceramics on resin cement Knoop Hardness Number. Oper. Dent..

[2] Ozturk E., Bolay S., Hickel R., Ilie N. (2013). Shear bond strength of porcelain laminate veneers to enamel, dentine and enamel-dentine complex bonded with different adhesive luting systems. J. Dent..

[3] Kilinc E., Antonson S.A., Hardigan P.C., Kesercioglu A. (2011). The effect of ceramic restoration shade and thickness on the polymerization of light- and dual-cure resin cements. Oper. Dent..

[4] Hooshmand T., Mohajerfar M., Keshvad A., Motahhary P. (2011). Microleakage and marginal gap of adhesive cements for noble alloy full cast crowns. Oper. Dent..

[5] Janda R., Roulet J.F., Latta M., Kaminsky M., Ruttermann S. (2007). Effect of exponential polymerization on color stability of resin-based filling materials. Dent. Mater..

[6] Knezevic A., Zeljezic D., Kopjar N., Tarle Z. (2008). Cytotoxicity of Composite Materials Polymerized with LED Curing Units. Oper. Dent..

[7] Ozturk E., Bolay S., Hickel R., Ilie N. (2015). Effects of ceramic shade and thickness on the micro-mechanical properties of a light-cured resin cement in different shades. Acta Odontol. Scand..

[8] Hadis M., Leprince J.G., Shortall A.C., Devaux J., Leloup G., Palin W.M. (2011). High irradiance curing and anomalies of exposure reciprocity law in resin-based materials. J. Dent..

[9] Selig D., Haenel T., Hausnerova B., Moeginger B., Labrie D., Sullivan B., Price R.B. (2015). Examining exposure reciprocity in a resin based composite using high irradiance levels and real-time degree of conversion values. Dent. Mater..

[10] Rueggeberg F.A., Caughman W.F., Curtis J.W. (1994). Effect of light intensity and exposure duration on cure of resin composite. Oper. Dent..

[11] Fan P.L., Schumacher R.M., Azzolin K., Geary R., Eichmiller F.C. (2002). Curing-light intensity and depth of cure of resin-based composites tested according to international standards. J. Am. Dent. Assoc..

[12] Barghi N., Berry T., Hatton C. (1994). Evaluating Intensity Output of Curing Lights in Private Dental Offices. J. Am. Dent. Assoc..

[13] Almeida J.R., Schmitt G.U., Kaizer M.R., Boscato N., Moraes R.R. (2015). Resin-based luting agents and color stability of bonded ceramic veneers. J. Prosthet. Dent..

[14] Al-Dwairi Z.N., Alkhatatbeh R.M., Baba N.Z., Goodacre C.J. (2019). A comparison of the marginal and internal fit of porcelain laminate veneers fabricated by pressing and CAD-CAM milling and cemented with 2 different resin cements. J. Prosthet. Dent..

[15] Sener I., Turker B., Valandro L.F., Ozcan M. (2014). Marginal gap, cement thickness, and microleakage of 2 zirconia crown systems luted with glass ionomer and MDP-based cements. Gen. Dent..

[16] Flury S., Lussi A., Hickel R., Ilie N. (2013). Light curing through glass ceramics with a second- and a third-generation LED curing unit: Effect of curing mode on the degree of conversion of dual-curing resin cements. Clin. Oral. Investig..

[17] Watanabe H., Kazama R., Asai T., Kanaya F., Ishizaki H., Fukushima M., Okiji T. (2015). Efficiency of dual-cured resin cement polymerization induced by high-intensity LED curing units through ceramic material. Oper. Dent..

[18] Morimoto S., Zanini R.A.M., Meira J.B.C., Agra C.M., Calheiros F.C., Nagase D.Y. (2016). Influence of physical assessment of different light-curing units on irradiance and composite microhardness top/bottom ratio. Odontology.

[19] Tango R.N., Sinhoreti M.A., Correr A.B., Correr-Sobrinho L., Henriques G.E. (2007). Effect of Light-Curing Method and Cement Activation Mode on Resin Cement Knoop Hardness. J. Prosthodont..

[20] Bragança G.F., Vianna A.S., Neves F.D., Price R.B., Soares C.J. (2020). Effect of exposure time and moving the curing light on the degree of conversion and Knoop microhardness of light-cured resin cements. Dent. Mater..

[21] Peutzfeldt A., Lussi A., Flury S. (2016). Effect of High-Irradiance Light-Curing on Micromechanical Properties of Resin Cements. Biomed. Res. Int..

[22] Cadenaro M., Navarra C.O., Antoniolli F., Mazzoni A., Di Lenarda R., Rueggeberg F.A., Breschi L. (2010). The effect of curing mode on extent of polymerization and microhardness of dual-cured, self-adhesive resin cements. Am. J. Dent..

[23] Hooshmand T., Mahmoodi N., Keshvad A. (2009). Microhardness of a resin cement polymerized by light-emitting diode and halogen lights through ceramic. J. Prosthodont..

[24] Watts D.C. (2005). Reaction kinetics and mechanics in photo-polymerised networks. Dent. Mater..

[25] Pilo R., Folkman M., Arieli A., Levartovsky S. (2018). Marginal Fit and Retention Strength of Zirconia Crowns Cemented by Self-adhesive Resin Cements. Oper. Dent..

[26] Peroz I., Mitsas T., Erdelt K., Kopsahilis N. (2019). Marginal adaptation of lithium disilicate ceramic crowns cemented with three different resin cements. Clin. Oral. Investig..

[27] Tongtaksin A., Leevailoj C. (2017). Battery Charge Affects the Stability of Light Intensity from Light-emitting Diode Light-curing Units. Oper. Dent..

[28] Sadeghyar A., Watts D.C., Schedle A. (2020). Limited reciprocity in curing efficiency of bulk-fill resin-composites. Dent. Mater..

[29] Palagummi S.V., Hong T., Wang Z., Moon C.K., Chiang M.Y.M. (2020). Resin viscosity determines the condition for a valid exposure reciprocity law in dental composites. Dent. Mater..

[30] Musanje L., Darvell B.W. (2003). Polymerization of resin composite restorative materials: Exposure reciprocity. Dent. Mater..

[31] Peutzfeldt A., Asmussen E. (2005). Resin composite properties and energy density of light cure. J. Dent. Res..

[32] Dewaele M., Asmussen E., Peutzfeldt A., Munksgaard E., Benetti A., Finné G., Leloup G., Devaux J. (2009). Influence of curing protocol on selected properties of light-curing polymers: Degree of conversion, volume contraction, elastic modulus, and glass transition temperature. Dent. Mater..

[33] Asmussen E., Peutzfeldt A. (2005). Polymerization contraction of resin composite vs. energy and power density of light-cure. Eur. J. Oral Sci..

[34] Leprince J.G., Hadis M., Shortall A.C., Ferracane J.L., Devaux J., Leloup G., Palin W.M. (2011). Photoinitiator type and applicability of exposure reciprocity law in filled and unfilled photoactive resins. Dent. Mater..

